# Holiday Items

**Published:** 1911-01

**Authors:** 


### HOLIDAY ITEMS

Calox, the oxygen tooth powder,—the powder that cleanses the teeth and purifies the mouth,—is just the thing for a holiday present, because it makes a useful gift that will prove a lasting friend to him or her who receives, and renders happy him or her who gives. Calox not only ensures cleanliness but renders the mouth and teeth germ-free, while at the same time it prevents decay. Moreover, it is harmless, leaving the structures of the mouth wholesome, sweet, and odorless. If you have not tried it, now is the time to do so.

---

Apollinaris still remains the queen of table waters, notwithstanding the countless rivals that have appeared within the last dozen years or so. It is the only appropriate water to serve at banquets or large dinners, for which purpose it stands unrivaled. For the sickroom, too, it is unsurpassed, making an agreeable, refreshing drink, as well as an excellent remedy in many affections.

---

Moet and Chandon's White Seal Champagne is a delightful dinner beverage, and is a suitable present to make during this season of good cheer. Besides, it is one of the purest of sparkling wines, made from selected grapes, grown on a proper soil, hence is adapted for use by the sick whenever such a remedy is indicated. A cork tap keeps it fresh, even when used only by the teaspoonful, and also prevents waste, thus rendering its use economical.

